# Veno-Venoarterial Extracorporeal Membrane Oxygenation for Septic Cardiomyopathy Caused by Invasive Pneumococcal Infection: A Case Report

**DOI:** 10.7759/cureus.70463

**Published:** 2024-09-29

**Authors:** Shinya Iwase, Nobuya Kitamura, Kuniyuki Kako, Takuya Fusada, Foad Kheirandish, Yushi Shinozaki

**Affiliations:** 1 Department of Emergency and Critical Care Medicine, Kimitsu Chuo Hospital, Chiba, JPN

**Keywords:** extracorporeal membrane oxygenation, hybrid ecmo, icu, septic cardiomyopathy, septic shock

## Abstract

The efficacy of extracorporeal membrane oxygenation (ECMO) for circulatory support in septic shock, especially hybrid ECMO, remains uncertain. A 30-year-old woman presented with septic shock caused by invasive pneumococcal infection, requiring intensive care unit (ICU) admission. Despite maximal respiratory support, her condition worsened with a partial pressure of oxygen (PaO_2_)/fraction of inspired oxygen (FiO_2_) ratio < 60 (The ratio is an indicator of respiratory oxygenation), indicating severe hypoxia and requiring the initiation of veno-venous (V-V) ECMO within three hours. Progressive circulatory failure followed, marked by reduced cardiac function indicative of septic cardiomyopathy; septic cardiomyopathy is a reversible myocardial dysfunction that occurs in patients with sepsis. Transition to veno-venoarterial (V-VA) ECMO took place 13 hours after admission. Liberation from veno-arterial (V-A) ECMO on day 9 and V-V ECMO on day 16 paralleled improvements in circulatory and respiratory functions. Necrosis of both lower extremities, pneumonia and bloodstream infection caused by *Pseudomonas aeruginosa*, prolonged ICU stay until discharge on day 52. Weaned off the ventilator, and with fully recovered consciousness and cardiac function, she was transferred to a rehabilitation facility on day 89. At follow-up more than six months after disease onset, she was doing well and continuing rehabilitation. This case enhances our understanding that when septic cardiomyopathy causes circulatory failure early in the treatment of septic shock, ECMO can serve as life-saving circulatory support. Additionally, the successful use of V-VA ECMO in this case highlights its potential as a therapeutic strategy for patients with severe respiratory failure complicated by septic cardiomyopathy, especially those initially managed with V-V ECMO. Timely transition to V-VA ECMO may improve outcomes in septic patients receiving V-V ECMO when cardiac dysfunction worsens due to septic cardiomyopathy.

## Introduction

Extracorporeal membrane oxygenation (ECMO) provides life-support for patients whose heart and lungs cannot function properly. The two main ECMO configurations are veno-venous (V-V) ECMO for severe respiratory failure and veno-arterial (V-A) ECMO, for both cardiac and respiratory failure. In addition to these two basic configurations, when respiratory and cardiac failure coexist, V-V and V-A ECMO can be combined using an additional cannula to divide return blood flow, a configuration known as “hybrid” ECMO [1]. As for the term of the hybrid ECMO, the term "veno-venoarterial (V-VA)" ECMO refers to adding V-A to V-V ECMO, while "veno-arteriovenous (V-AV)" ECMO denotes adding V-V to V-A ECMO, although these terms are often conflated [2].

In sepsis, V-V ECMO is recommended for severe acute respiratory distress syndrome (ARDS) when conventional mechanical ventilation fails, though outcomes for septic patients on V-A ECMO are inconsistent [3-5]. The latest revision of the Surviving Sepsis Campaign Guideline (SSCG), the international practice guideline for sepsis, recommends V-V ECMO for severe ARDS (weak recommendation, low quality of evidence), but does not address the use of V-A ECMO for circulatory failure [6].

Circulatory failure in sepsis arises from not only distributive shock due to vascular paralysis and capillary leakage but also from cardiogenic shock caused by septic cardiomyopathy. Failure to distinguish between these two causes of circulatory failure may explain the inconsistent effects of V-A ECMO in sepsis across previous studies. Although no universally accepted definition or diagnostic criteria for septic cardiomyopathy exist, it is a reversible myocardial disorder driven by inflammatory mediators such as endotoxin, tumor necrosis factor-alpha (TNF-α), and interleukin-1-beta (IL-1β) [7]. The pathophysiology of septic cardiomyopathy remains unclear; however, it is believed that inflammatory mediators impair cardiac contractility and induce mitochondrial dysfunction, which contributes to cardiac failure [7,8]. Myocardial damage in septic cardiomyopathy is typically reversible, with improvement observed within 7-10 days [7]. A key clinical finding is the reduction in left ventricular ejection fraction (LVEF) on echocardiography. The reported prevalence of septic cardiomyopathy ranges from 10% to 70% [8], likely due to the absence of standardized diagnostic criteria. Additionally, septic cardiomyopathy has been significantly associated with increased in-hospital mortality [9]. However, given its reversible nature, septic patients with septic cardiomyopathy may benefit from V-A ECMO since the primary cause of circulatory failure in such patients is septic cardiomyopathy. Recently, some studies have shown that V-A ECMO improves outcomes in patients with septic cardiomyopathy [10,11]. It is crucial to differentiate circulatory failure in septic patients and identify septic cardiomyopathy, a condition for which ECMO may be beneficial in improving patient outcomes.

Studies on the utility of V-VA ECMO for septic patients are limited to case reports and small case series [12,13]. Notably, only one study has specifically examined the use of V-VA ECMO in patients with septic cardiomyopathy [14]. That study suggested that V-VA ECMO may provide potential benefits for patients with septic cardiomyopathy. However, there is no clear guidance on optimal patient selection, timing of ECMO initiation, or management. Long-term outcomes and quality of life (QOL) in septic patients treated with V-VA ECMO also remain unclear.

This report describes a case of septic shock due to an invasive pneumococcal infection, effectively managed with V-VA ECMO for progressive circulatory failure caused by septic cardiomyopathy, after the initiation of V-V ECMO for respiratory failure. The reasons for choosing V-VA ECMO over V-A ECMO in this case are described in the case presention section. This case provides valuable insights for intensivists on managing complex septic shock with advanced ECMO techniques, particularly the timing and management of V-VA ECMO initiation, as well as considerations for long-term outcomes and QOL in septic patients who receive V-VA ECMO.

## Case presentation

Initial presentation

**Figure 1:**
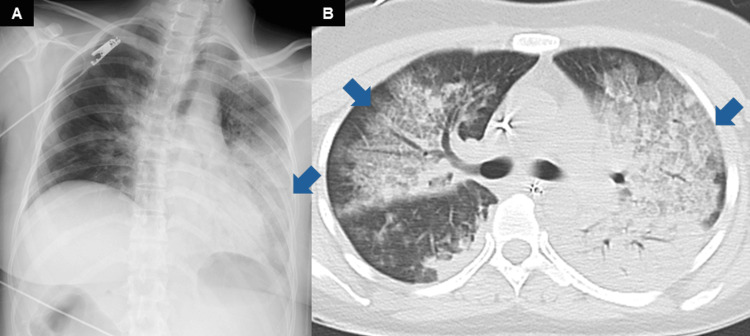
Chest X-ray (A) and computed tomography (B) at emergency department admission

A 30-year-old woman presented to the referral hospital with fever and respiratory distress that began that day. Her temperature was 38°C and oxygen saturation remained 85% despite receiving 15 L/minute of oxygen. She was subsequently referred to our hospital. Upon arrival, she exhibited signs of respiratory failure with a respiratory rate of 40 breaths per minute, along with circulatory failure indicated by an unmeasurable blood pressure. Chest X-ray and computed tomography (CT) scans revealed consolidation with air bronchograms in the left lower lobe and bilateral ground-glass opacities (Figure 1). Echocardiography indicated normal cardiac function with an LVEF of 60%. *Streptococcus pneumoniae* antigen was positive in urine, and gram-positive diplococci were observed in a sputum Gram stain; later, blood cultures also detected *S. pneumoniae*. Septic shock caused by invasive pneumococcal infection was confirmed with positive *S. pneumoniae* antigen in urine, gram-positive diplococci in sputum, and *S. pneumoniae* isolated from blood cultures. She was intubated, placed on mechanical ventilation, and admitted to the intensive care unit (ICU).

ECMO management

Respiratory status declined with a partial pressure of oxygen (PaO_2_)/fraction of inspired oxygen (FiO_2_) ratio < 60 (mechanical ventilator settings: FiO_2_ 1.0, positive end-expiratory pressure (PEEP) 14 cmH_2_O), necessitating the initiation of V-V ECMO three hours post-ICU admission using a MERA centrifugal blood pump system (Senko Medical Instrument Mfg. Co., Ltd, Tokyo, Japan). A 24 Fr drainage cannula and a 20 Fr return cannula were percutaneously inserted into the right femoral vein and internal jugular vein, respectively. V-V ECMO was initiated with a blood flow of 3.5 L/minute. Subsequently, however, blood pressure dropped, and echocardiography showed reduced cardiac contractility (LVEF 10%), which was normal at the time of presentation, indicating septic cardiomyopathy.

**Figure 2:**
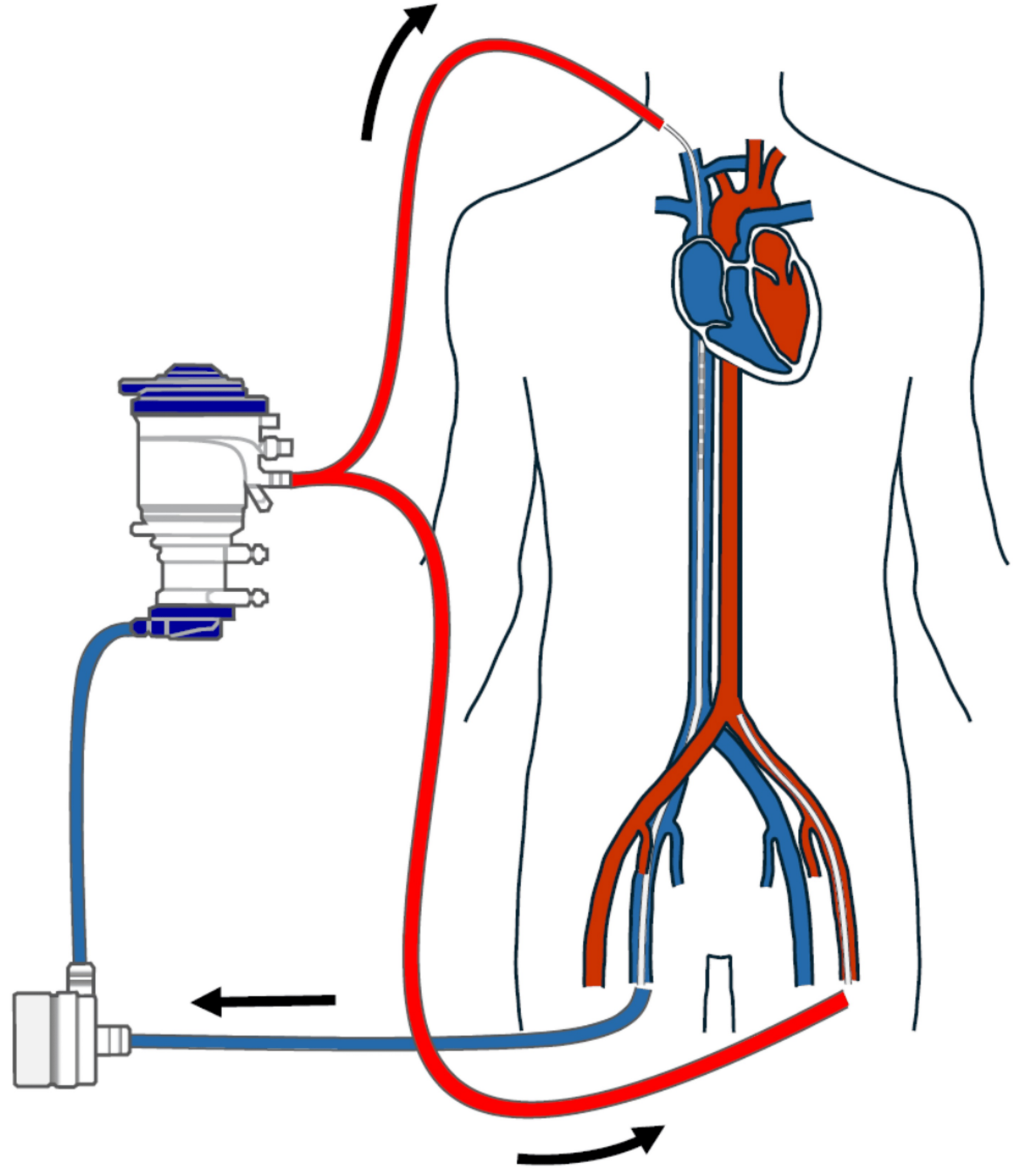
Veno-venoarterial ECMO circuit Venous blood is drained through a catheter in the right femoral vein and pumped to an artificial lung for oxygenation and carbon dioxide removal. The oxygenated blood is then split via a Y-shaped connector, with one portion returning to the venous system through the right internal jugular vein, while the other is directed to the arterial system via the left femoral artery. ECMO: extracorporeal membrane oxygenation Image Credit: Authors

Despite receiving noradrenaline at 1.0 µg/kg/minute, vasopressin at 0.03 units/minute, and dobutamine at 5 µg/kg/minute, her mean arterial pressure remained at 50 mmHg, and blood lactate levels increased to 7.4 mmol/L. As she was unable to maintain hemodynamic stability with vasoactive drugs alone, we decided to transition from V-V to V-VA ECMO. We opted for V-VA ECMO instead of V-A ECMO in this case due to the following considerations: (i) she was already on V-V ECMO to manage respiratory failure; (ii) the severity of the respiratory failure suggested that V-VA ECMO would provide more stable oxygenation compared to V-A ECMO; and (iii) it was expected that her respiratory recovery would be prolonged, requiring a later transition back to V-V ECMO. A 20 Fr return cannula was inserted into the left femoral artery, converting from V-V to V-VA ECMO 13 hours post ICU admission (Figure 2).

**Figure 3:**
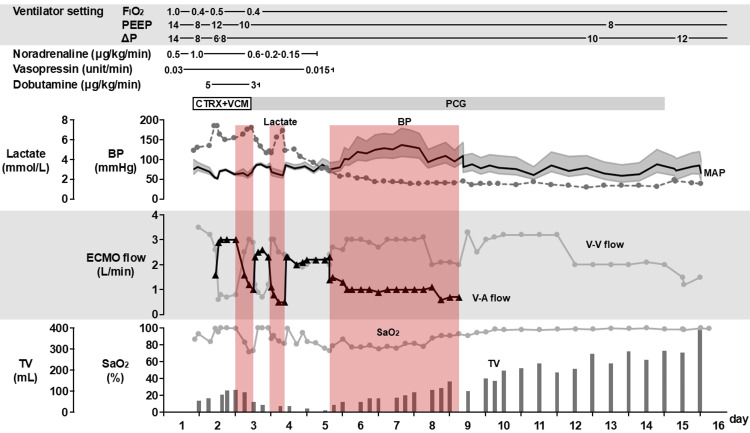
Management of ECMO and the associated respiratory and circulatory parameters PEEP: positive end expiratory pressure^*^; ΔP: driving pressure^†^; CTRX: ceftriaxone; VCM: vancomycin; PCG: penicillin G; BP: blood pressure; MAP: mean arterial pressure; ECMO: extracorporeal membrane oxygenation; TV: tidal volume; SaO_^2^_, arterial oxygen saturation ^*^A mechanical ventilator setting that helps maintain lung expansion by applying a small amount of pressure after exhalation, aiding in respiratory oxygenation. ^†^The difference between plateau pressure (the pressure in the lungs at the end of inhalation) and PEEP. It reflects the pressure required to expand the lungs with each breath.

Figure 3 shows the clinical course from ICU admission to ECMO weaning. Initially, no pulse pressure was detected, and V-VA ECMO was initiated with a V-A flow of 3.0 L/minute (total flow 3.8 L/minute) to maintain a mean arterial pressure > 65 mmHg. Hemodynamic stability relied completely on V-A ECMO, with arterial oxygen saturation (SaO_2_) of the right radial artery at 99% even with a V-V flow of 0.8 L/minute. On day 3, as pulse pressure increased, V-A flow was reduced to 1.0 L/minute, and V-V flow was adjusted to 3.0 L/minute. However, blood lactate levels began to rise, and SaO_2_ dropped below 70%, necessitating a return to V-A-based ECMO management (V-A flow 2.3-2.5 L/minute, V-V flow 0.8-1.2 L/minute). On day 4, with a reduction in vasoactive drugs, V-A flow was decreased again, and V-V-based ECMO management resumed. Despite SaO_2_ remaining above 80%, blood lactate levels continued to rise, prompting an increase in V-A flow and setting ECMO management at V-A:V-V = 1:1 (both V-A and V-V flows at 2.0-2.3 L/minute). Subsequently, vasoactive agents were discontinued, and V-A flow was reduced to 1.0 L/minute on day 5. Blood lactate levels began to decrease, and V-V-based ECMO management was maintained (V-A flow 1.0 L/minute, V-V flow 3.0 L/minute). On day 9, V-A ECMO was withdrawn, and V-V ECMO (flow 3.3 L/minute) continued. The respiratory condition improved, and V-V ECMO was weaned off on day 16.

Complications

Due to necrosis of both lower legs, pneumonia, and bloodstream infection caused by *Pseudomonas aeruginosa*, she required extended ICU treatment. Initial treatment with tazobactam/piperacillin (TAZ/PIPC) was started on day 17 but failed to control the infection, leading to wound infections in the necrotic areas of both legs. To save her life, bilateral lower limb amputations were performed on day 23 as a source control measure. Following the surgery, a three-week course of antimicrobial therapy was administered until day 37, successfully controlling the *P. aeruginosa* infection. Despite this, mechanical ventilation was still required, and a tracheotomy was performed on day 28.

Recovery

**Figure 4:**
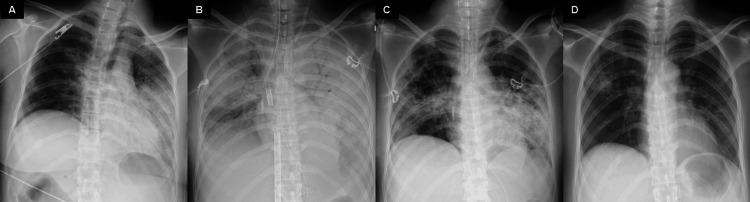
Changes in chest X-ray findings The lung consolidation, initially confined primarily to the left side at admission (A), had significantly worsened bilaterally by the time ECMO was initiated (B). By the time the patient was weaned off ECMO, consolidation in the bilateral lung fields had significantly improved (D), with residual consolidation limited to the right middle and left middle and lower lung fields. At discharge, after the patient had been weaned off the ventilator and no longer required oxygen, the chest X-ray findings were nearly normal (D). ECMO: extracorporeal membrane oxygenation

**Figure 5:**
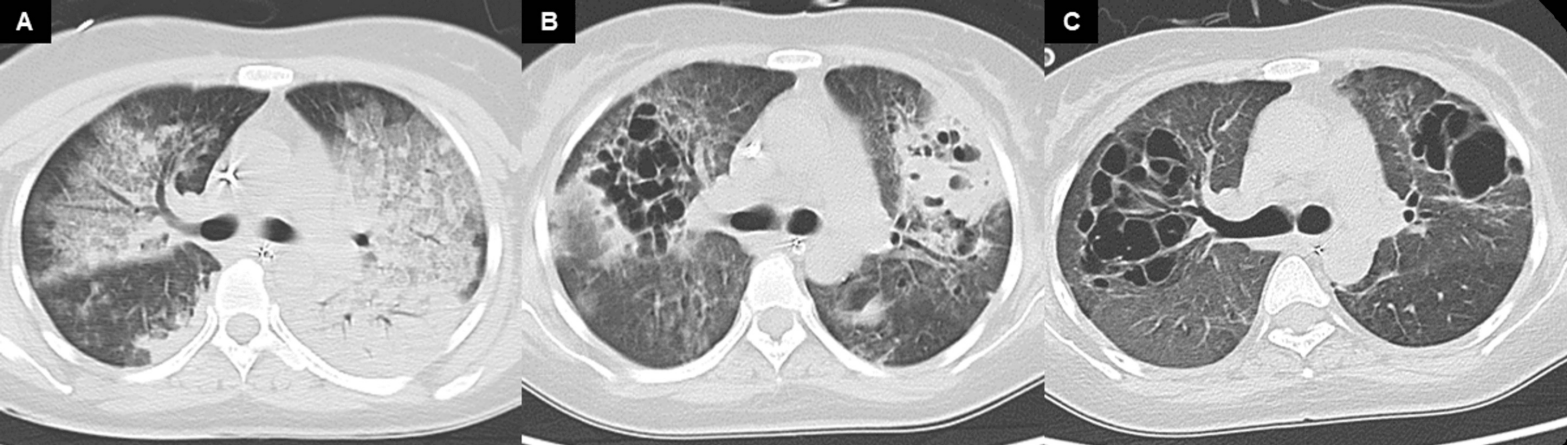
Changes in chest CT findings (A) On admission, CT scans revealed consolidation in the left lower lobe and bilateral GGO. (B) By the time of ECMO weaning, the GGO had resolved, but bilateral cavitary lesions from alveolar destruction and localized consolidation in the left lung were observed. (C) At ICU discharge, the cavitary lesions remained, though the consolidation had resolved. ECMO: extracorporeal membrane oxygenation; GGO: ground-glass opacities

The patient was discharged from the ICU on day 52 and weaned from the ventilator on day 53. Subsequently, she resumed oral intake, and the tracheostomy tube was removed. Chest X-ray and CT scans showed improvement in lung field consolidation (Figures 4, 5). Cardiac function fully recovered (LVEF 60%), and she regained full consciousness. She was transferred to a rehabilitation facility on day 89.

## Discussion

This case involved septic shock from invasive pneumococcal infection, initially treated with V-V ECMO for respiratory failure and later transitioned to V-VA ECMO due to worsening circulatory failure from septic cardiomyopathy, resulting in a successful recovery.

Use of V-A ECMO in septic cardiomyopathy

Circulatory failure in sepsis encompasses both distributive shock and cardiogenic shock from septic cardiomyopathy [15]. Septic cardiomyopathy is a reversible myocardial disorder, typically recovering within a week [7]. Therefore, V-A ECMO can be valuable supportive therapy until recovery. Recent studies have shown the efficacy of V-A ECMO in septic shock patients with impaired cardiac function [10,11]. Historically, ECMO as a form of circulatory support in sepsis was sometimes viewed as contraindicated [15]. However, by differentiating the underlying causes of circulatory failure, a patient population has emerged that could benefit from ECMO in this context: those with septic cardiomyopathy. In our patient, the LVEF, initially normal, decreased after starting V-V ECMO. The patient was transitioned to V-VA ECMO when septic cardiomyopathy was identified as the cause of the hemodynamic deterioration. Vasoactive drugs were reduced from day 3, and the patient was weaned off V-A ECMO on day 9, fully recovering cardiac function.

The role of hybrid ECMO in sequential respiratory and circulatory failure

When respiratory and circulatory failures coexist, hybrid ECMO, which combines V-V and V-A ECMO with an additional third cannula to divide return blood flow, is considered. Hybrid ECMO may provide more effective oxygenation and hemodynamic support than V-A ECMO by returning blood to both the arterial and venous systems [15]. A hybrid ECMO that begins with a V-V configuration is called V-VA ECMO, while one that starts with a V-A configuration is referred to as V-AV ECMO [2]. Although both ultimately lead to the same final configuration, it is essential to distinguish between them, as they indicate different underlying pathologies depending on whether the patient initially presents with respiratory failure or circulatory failure. In the case presented here, V-V ECMO was initiated for respiratory failure, then converted to V-VA ECMO for circulatory failure due to septic cardiomyopathy following respiratory failure. The decision to use V-VA ECMO in the case was based on the following: (i) V-V ECMO had already been performed before V-A ECMO, (ii) respiratory failure was so severe that V-A ECMO alone could not provide adequate oxygenation, and (iii) respiratory recovery was expected to take longer than circulatory recovery, necessitating the continuation of V-V ECMO.

Survival outcomes and case comparison in hybrid ECMO for septic cardiomyopathy

The survival rate of patients on hybrid ECMO ranges from 39% to 75% [14,16-18] (Table 1). This variability may be attributed to studies not distinguishing between V-VA and V-AV ECMO and inconsistencies in the underlying conditions of the patient cohorts. Studies that did not differentiate between V-VA and V-AV ECMO reported survival rates of 39% to 42.9% [16,17]. In a study involving ARDS patients with circulatory failure, 79% of whom were septic, the survival rate was 52% [18]. Conversely, retrospective, single-center case series by Vogel et al. focusing solely on patients requiring V-VA ECMO due to septic cardiomyopathy, like the case presented here, reported a 75% survival rate [14], surpassing those in the three prior studies [16-18]. In Vogel et al.'s study, 12 patients with severe respiratory failure and septic cardiomyopathy were treated with V-VA ECMO [14]. Five of these patients experienced cardiac arrest and required cardiopulmonary resuscitation before ECMO initiation. Ten patients had pneumonia, three of which were caused by *S. pneumoniae*, like our case. The median LVEF at the start of V-VA ECMO was 16.25% (10% in our case), with survivors recovering to an LVEF of 55-60% (60% in our case). The course of LVEF recovery was consistent with our case. The median duration of V-VA ECMO was four days (eight days in our case), with a median total ECMO duration of nine days (16 days in our case), a median ICU stay of 21 days (52 days in our case), and a median hospital stay of 62 days (89 days in our case). In our case, the ECMO duration, ICU stay, and hospital stay were relatively longer. The V-VA ECMO duration could potentially have been shortened, as the patient maintained circulatory stability for more than three days with approximately 1.0 L/minute of V-A flow. The extended duration of V-V ECMO, ICU stay, and hospital stay in our case was likely influenced by complications from secondary *P. aeruginosa* infection and bilateral lower extremity amputations.

**Table 1:** Summary of previous reports on hybrid ECMO ARDS: acute respiratory distress syndrome; PE: pulmonary embolism; IPF: idiopathic pulmonary fibrosis; HF: heart failure; ECMO: extracorporeal membrane oxygenation; V-VA: veno-venoarterial; V-AV: veno-arteriovenous

Author (year)	Type of study	Underlying disease	V-VA, n	V-AV, n	Initially hybrid, n	Survival
Biscotti et al., 2014 [16]	Single-center retrospective	ARDS, PE, IPF, etc.	8	2	11	42.9% (Hospital discharge)
Werner et al., 2016 [17]	Single-center retrospective	HF, ARDS, sepsis	6	7	10	39% (Hospital discharge)
Erlebach et al., 2022 [18]	Multi-center retrospective	ARDS (sepsis, 79%)	53	-	20	52% (ICU survival)
Vogel et al., 2018) [14]	Single-center retrospective	Respiratory failure with septic cardiomyopathy	12	-	-	75% (Hospital discharge

Considerations for future research

In our case, V-VA ECMO was lifesaving. However, in complex cases like this, ECMO alone is insufficient; a multidisciplinary approach, including appropriate antimicrobial therapy, fluid management, and ventilatory support, is essential. Despite our young patient with no significant medical history, secondary infection and bilateral lower extremity amputations significantly impacted her future QOL. Given the potential for serious complications even in a young, healthy patient, the use of V-VA ECMO in elderly patients should be approached with greater caution. Further research is needed to determine optimal patient selection such as stratifying patients based on age, infection focus, or causative organism. Additionally, biomarkers like lactate levels and clinical indicators, including refractory shock despite optimal vasopressor support and new-onset left ventricular dysfunction, may help identify those most likely to benefit from V-VA ECMO.

Complications

Lower extremity ischemia is a significant complication of V-A ECMO, particularly when femoral artery cannulation is used. Distal perfusion catheters are among the most effective preventive measures but in cases of irreversible ischemia, amputation of the lower extremity may be required [19]. Our patient had a distal perfusion catheter in place yet lower extremity ischemia still developed. While ECMO-related complications cannot be completely excluded as the cause of lower extremity necrosis in our patient, the necrosis was bilateral, affecting both legs, rather than localized to the left leg where a return cannula was placed in the femoral artery, indicating a substantial impact of invasive pneumococcal infection.

Clinical applications of V-VA ECMO in septic cardiomyopathy

Septic cardiomyopathy is a reversible condition that can improve quickly. When septic cardiomyopathy leads to circulatory failure in septic patients, using V-A ECMO may be beneficial. V-VA ECMO, though more complex than V-V or V-A ECMO, could be an effective treatment for complications of septic cardiomyopathy in patients initially treated with V-V ECMO, especially in centers with ECMO expertise.

## Conclusions

This report highlights the potential effectiveness of V-VA ECMO in saving the lives of patients with septic shock, characterized by severe respiratory failure and circulatory failure due to septic cardiomyopathy. Our patient, initially on V-V ECMO for respiratory failure, developed circulatory failure due to septic cardiomyopathy and was successfully managed by transitioning to V-VA ECMO. We also presented the management and withdrawal process of V-VA ECMO, along with the patient’s complications and recovery status at the time of discharge. In our case, a secondary *P. aeruginosa* infection extended the ICU stay, totaling 52 days, and necessitated long-term rehabilitation due to bilateral lower limb amputations, significantly impacting the patient's long-term QOL. Studies on V-VA ECMO for septic shock are currently limited, and the management, long-term prognosis, and QOL for patients receiving this treatment remain unclear. Our report aims to provide insights that could help address these gaps, though it is ultimately a single case report. Further research is needed to determine optimal patient selection criteria, potentially based on factors like age, infection source, causative organism, biomarkers such as lactate, and clinical signs like refractory shock and new-onset left ventricular dysfunction.

Managing V-VA ECMO is more complex than handling the basic configurations of V-V or V-A ECMO. It requires comprehensive patient assessment skills, as both V-V and V-A flows must be adjusted according to ongoing respiratory and circulatory evaluations. Healthcare providers must be capable of making swift decisions to modify the ECMO configuration in response to changing patient needs. Therefore, the indications for V-VA ECMO must be carefully evaluated in high-volume ECMO centers. Additionally, the indication for V-VA ECMO should be considered more carefully in elderly patients, as septic shock can pose serious risks even for younger individuals without prior medical history. The use of V-VA ECMO in septic shock is a promising approach that could reshape clinical practice. While studies are limited, our report highlights the need for further investigation to better understand the appropriate patient population, the management strategies, and the long-term outcomes. Exploring these areas could ultimately lead to more tailored interventions and improved survival rates for patients with septic shock.
